# Voice Change Following Third Molar Alveolectomy: A Rare and Preventable Complication

**DOI:** 10.3390/dj14040232

**Published:** 2026-04-14

**Authors:** Lara Majcen, Marko Tarle, Mario Raos, Josip Biočić, Berisav Perić, Davor Brajdić, Petar Đanić, Ivan Salarić

**Affiliations:** 1 Private Dental Practice, 10000 Zagreb, Croatia; laramajcen01@gmail.com; 2 Department of Maxillofacial Surgery, School of Dental Medicine, University of Zagreb, 10000 Zagreb, Croatia; mtarle@kbd.hr; 3 Department of Maxillofacial and Oral Surgery, University Hospital Dubrava, 10000 Zagreb, Croatia; biocic@sfzg.unizg.hr (J.B.); pericb@kbd.hr (B.P.); dbrajdic@kbd.hr (D.B.); salaric@sfzg.unizg.hr (I.S.); 4 Division of Oral Surgery, Dental Polyclinic Zagreb, 10000 Zagreb, Croatia; marioraos7@gmail.com; 5 Department of Oral Surgery, School of Dental Medicine, University of Zagreb, 10000 Zagreb, Croatia

**Keywords:** postoperative complications, tooth extraction, voice disorders, emphysema

## Abstract

**Background:** Emphysema is a rare complication of dental procedures. The highest incidence has been associated with the use of air turbine handpieces. If not recognized and treated promptly, this complication can lead to serious outcomes. **Methods:** We present a case of a 43-year-old man who developed deep cervicofacial and subcutaneous emphysema of the face, neck and chest along with the specific voice change due to nose blowing shortly after alveolectomy of the lower right third molar. **Results:** The patient was hospitalized, conservatively treated, monitored and treated with intravenous antibiotic therapy. After two weeks he made a full recovery. **Conclusions**: It is essential to recognize and adequately manage emphysema, and to inform patients postoperatively not to blow their nose after third molar alveolectomy to prevent this rare but potentially serious complication. To our knowledge, this is the first reported case of voice alteration caused by nasopharyngeal emphysema following third molar alveolectomy.

## 1. Introduction

Subcutaneous or submucosal emphysema is a relatively rare complication of dental procedures. It occurs when air enters soft tissues, resulting in distension of the skin or mucosa. Air may spread along fascial planes into the periorbital region, mediastinum, and into the pericardial and thoracic spaces, leading to swelling and crepitus [1,2,3]. Potential causes of cervicofacial subcutaneous emphysema include maxillofacial trauma, head and neck surgery, general anesthesia, endotracheal intubation and ventilation, Valsalva maneuver, gas-producing bacterial infections (e.g., *Clostridium perfringens*), and certain dental procedures [4]. The most frequent causes of emphysema during or after dental procedures are tooth preparation for prosthetic restorations and endodontic therapy, extractions and other oral surgical procedures, and the use of dental lasers and air turbines [1,5,6,7,8]. The majority of published case reports on the topic have been associated with the use of air turbines. These are powered by compressed air at 3.5–4.0 kg/cm^2^, rotating at approximately 450,000 revolutions per minute [1,4]. Water spray nozzles attached to the turbine tip help dissipate heat generated by friction and remove debris [4]. This compressed air can be forced into subcutaneous tissue and spread proximally from the extraction site. In addition to these causes, emphysema may also occur perioperatively after endotracheal intubation, tracheotomy or trauma. In most cases, emphysema resolves spontaneously within seven days with conservative treatment, which includes analgesics, antibiotic prophylaxis and close patient monitoring to prevent complications. Common complications include pneumothorax, air embolism, cardiac tamponade, mediastinitis, and Collet–Sicard syndrome [1,9]. While emphysema is a relatively rare complication, other common postoperative issues following third molar alveolectomy include pain, swelling, and trismus, especially if the medial pterygoid muscle is injured. Less frequent complications include alveolar infection, alveolar osteitis, damage to adjacent teeth, and injury to the inferior alveolar nerve [10].

We present a case of pharyngeal and subcutaneous emphysema of the face, neck, and chest following alveolectomy of the lower right third molar, with an unusual presentation of altered voice.

## 2. Case Report

**Figure 1:**
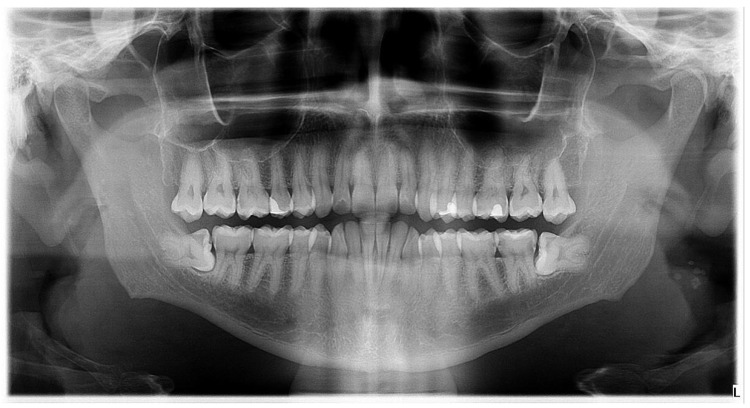
A panoramic X-ray taken before the alveolectomy of the lower right third molar.

A 43-year-old male was referred to the Oral Surgery Division, Department of Maxillofacial and Oral Surgery, Dubrava University Hospital, for alveolectomy of the lower right third molar (Figure 1). Medical history revealed Crohn’s disease, for which he was taking azathioprine (Imuran) and sulfasalazine (Salazopyrin).

Alveolectomy of the lower right third molar was performed under local anesthesia. A mucoperiosteal flap was elevated, and osteotomy was carried out using a physiodispenser with a surgical handpiece under continuous saline irrigation (without the use of an air-driven turbine). The crown was sectioned and removed first, followed by separate removal of the roots. The lingual cortical plate remained intact throughout the procedure. The surgical site was irrigated and sutured. The total duration of the procedure, including administration of local anesthesia, was approximately 30 min. The patient was given instructions on analgesic use and on how to obtain oral hygiene and clean the sutures. Unfortunately, he had not been instructed to avoid nose blowing.

**Figure 2:**
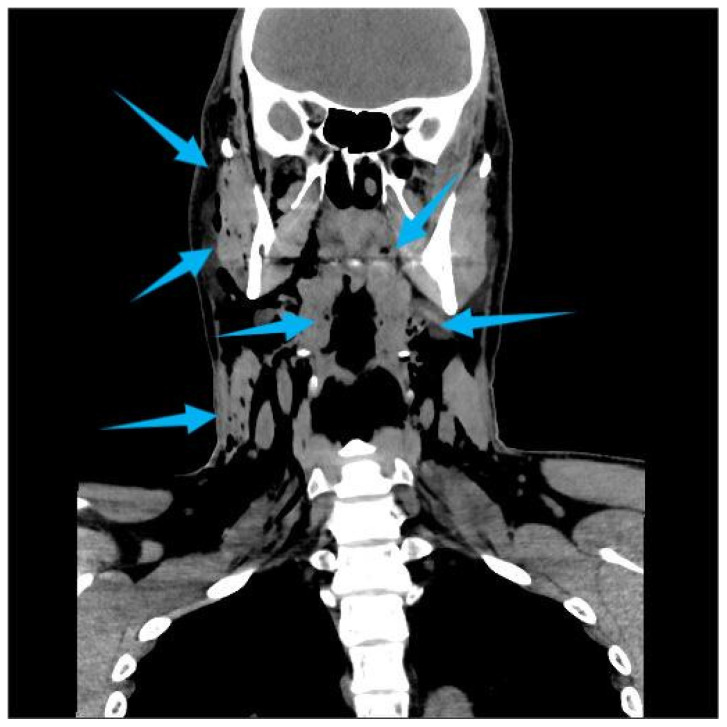
Coronal Multislice Computed Tomography (MSCT) image demonstrating extensive subcutaneous emphysema of the right side of the face, involving cervical soft tissues bilaterally and extending bilaterally into the upper thoracic region. The arrows highlight the presence of emphysema.

**Figure 3:**
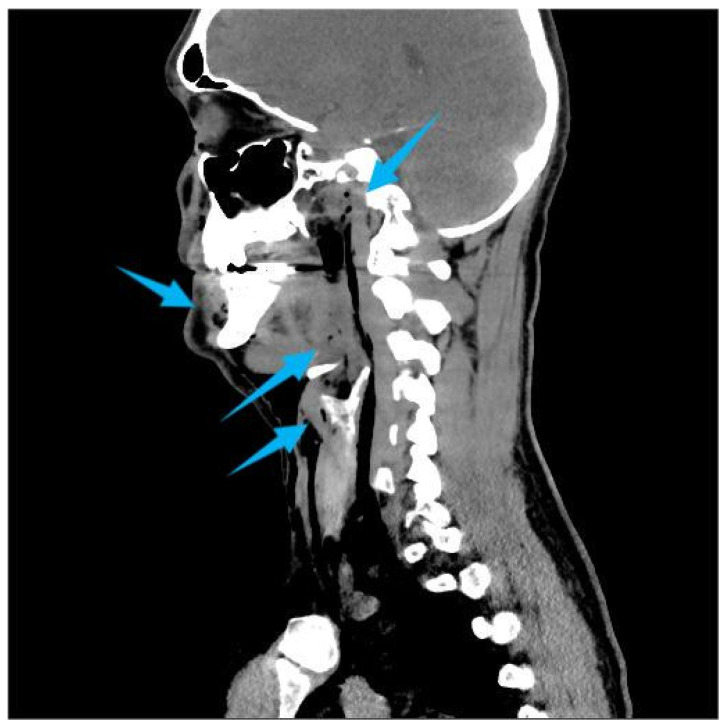
Sagittal Multislice Computed Tomography (MSCT) image demonstrating submucosal emphysema of the nasopharynx. The arrows highlight the presence of emphysema.

**Figure 4:**
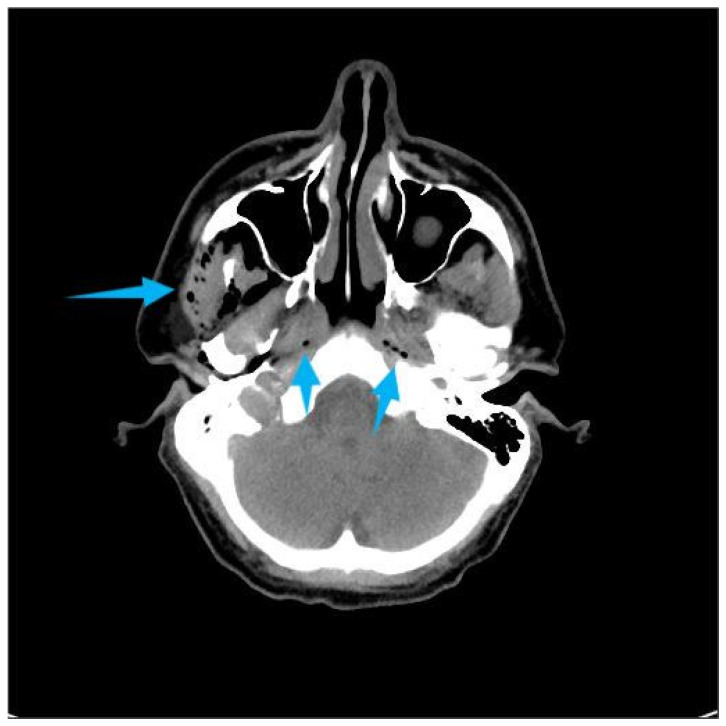
Axial Multislice Computed Tomography (MSCT) image showing a submucosal emphysema of the pharynx and a subcutaneous emphysema of the right side of the face. The arrows highlight the presence of emphysema.

**Figure 5:**
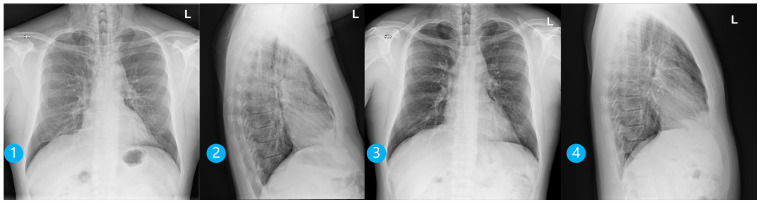
Posteroanterior and lateral chest radiographs taken on the day of emphysema occurrence (**1**,**2**) and five days later (**3**,**4**). In (**1**,**2**), subcutaneous emphysema of the soft tissues of the neck and upper thorax bilaterally is observed, along with pneumomediastinum. In (**3**,**4**), a regression of subcutaneous emphysema in the soft tissues of the neck and upper thorax bilaterally, along with pneumomediastinum, can be observed.

One hour after the procedure, the patient returned complaining of facial swelling that developed immediately after nose blowing. Clinical examination revealed palpable crepitus, suggesting emphysema. The patient was advised to rest and was prescribed oral amoxicillin 500 mg three times daily. Later the same day, he presented to the emergency department due to an altered voice and a sensation of air accumulating beneath the skin of the neck and chest. There were no signs of respiratory distress or fever. His blood pressure was 140/75 mmHg, pulse 80 bpm, and body temperature 36.3 °C. Clinical signs included hoarseness, “Donald Duck-like” voice, palpable emphysema intraorally on the right buccal side, and extraoral emphysema in the soft tissues of both cheeks and neck extending caudally to the jugular notch and periclavicular region, without erythema. Fiberoptic examination confirmed normal vocal cord mobility. Computed Tomography (CT) imaging revealed extensive air in the retropharyngeal and nasopharyngeal space, subcutaneous adipose tissue on the right side of the face, bilaterally in the neck, and in the upper thoracic wall (Figure 2, Figure 3 and Figure 4). C-reactive protein (CRP) was elevated at 23 mg/L, while other hematological and biochemical parameters were within normal limits. The patient was hospitalized and treated with intravenous amoxicillin–clavulanic acid 1.2 g, three times daily. Clinical and radiological findings improved during hospitalization, and the patient was discharged after five days. Posteroanterior and lateral chest radiographs were taken on the day of the hospitalization and on the day of discharge (Figure 5). The latter showed the regression of the subcutaneous emphysema in the soft tissues of the neck and upper thorax bilaterally and the pneumomediastinum. Recovery of the voice was observed gradually and the patient reported full voice recovery eight days after alveolectomy.

The patient was instructed to continue antibiotics for a total of seven days, maintain oral hygiene, and rinse with 0.12% chlorhexidine solution three times daily for seven days. A follow-up examination was conducted one week later and a full recovery noted (Table 1).

## 3. Discussion

Subcutaneous and deep cervicofacial emphysema is a rare complication of dental procedures, most often occurring during extractions and endodontic treatments [11]. It may also be triggered by coughing, vomiting, smoking, forceful nose blowing, sneezing, playing wind instruments, or activities that increase intrathoracic pressure [12]. Two conditions must be met for emphysema to develop: communication between the oral cavity and soft tissue, and the introduction of pressurized air [3]. Spille et al. published a systematic review of the literature from 2010 to 2021, identifying 33 case reports of emphysema caused by dental procedures, extending from the face to the mediastinum. In 63.6% of cases, the procedures involved only mandibular teeth, predominantly molars. In 48.5% of cases, teeth were extracted, while the remainder (51.5%) involved restorative, endodontic, or prosthetic treatments. Most patients (81.8%) received intravenous antibiotic therapy [5]. In our case, nose blowing led to air spreading from the alveolar socket to the submandibular space and then into pterygomandibular space. The air then extended through the posterior margin of the medial pterygoid muscle into the parapharyngeal space, and further into the nasopharyngeal and retropharyngeal spaces. Narrowing of the nasopharynx and pressure on the Eustachian tube altered airflow and resulted in voice changes (“Donald Duck-like” speech). The retropharyngeal space, separated from the parapharyngeal space by the buccopharyngeal fascia, extends from the base of the skull to the C7 or T1 vertebrae, where the buccopharyngeal and alar fasciae converge. Laterally, it is bordered by the carotid sheath. Descending air can pass from the retropharyngeal space between the alar and prevertebral fasciae to the diaphragm and posterior mediastinum, potentially leading to mediastinitis [13]. Air in the mediastinum can cause vasodilation and hypotension, hypercapnia, acidosis, and air embolism [11]. Clinical signs suggestive of pneumomediastinum include dyspnea with a brassy voice, chest or back pain, and Hamman’s sign (mediastinal crepitus synchronous with the heartbeat) [4,14]. Fortunately, air did not spread caudal from the retropharyngeal space. Symptoms and signs of subcutaneous emphysema may vary. In some patients, swelling occurs suddenly, while in others, it develops gradually over hours. Peters et al. reviewed 26 reported cases and found that 96% presented with crepitus, 80% with swelling, and a smaller number reported neck or chest pain. In most cases, air spread to the periorbital region, face, and neck, and 77% of patients developed pneumomediastinum [15]. Our patient also had a subcutaneous emphysema caused by air spreading from the submandibular space underneath the skin of the face, neck and chest.

Our patient took azathioprine and sulfasalazine for Crohn’s disease, and while they may modestly influence immune response, their effect on routine oral wound healing (e.g., after a tooth alveolectomy) is generally limited and not clinically significant in well-controlled patients. Routine perioperative antibiotic coverage is not generally necessary for tooth alveolectomy in patients taking azathioprine and sulfasalazine, provided the patient is otherwise clinically stable (no severe immunosuppression, no neutropenia, and good oral hygiene). Our patient’s blood count, obtained by his gastroenterologist on a routine check-up one day prior to alveolectomy, was within normal limits and therefore we did not consider prescribing antibiotics immediately after our procedure, i.e., prior to the development of emphysema. Antibiotics may be considered on a case-by-case basis if additional risk factors are present, such as high-dose or combination immunosuppression (e.g., with corticosteroids or biologics), documented leukopenia/neutropenia, poorly controlled systemic disease, and extensive or prolonged surgery with higher infection risk (which was not the case for our patient) [16].

It is important to differentiate emphysema from other complications that cause soft tissue swelling, such as hematoma, allergic reaction, or angioedema [4]. In our case, cheek swelling and discomfort occurred immediately, followed by voice changes several hours later. To our knowledge, this is the first reported case of deep cervicofacial emphysema with altered voice following lower third molar alveolectomy. Although rare, complications such as pneumothorax, air embolism, mediastinitis, cranial nerve paralysis, and cardiac tamponade can also occur [1,9]. Unlike the case reported by North et al. where vocal fold paresis occurred, our patient’s vocal changes were due to nasopharyngeal narrowing without nerve injury [9]. In most cases, emphysema resolves spontaneously within 2 to 10 days without complications. Treatment is therefore usually symptomatic. Cold compresses are recommended on the first day to prevent air spread, followed by warm compresses to improve circulation and promote air resorption. Intravenous antibiotics are advised to prevent infection-related complications, along with analgesics if needed, airway monitoring, and general clinical observation [17]. Successful postoperative recovery after third molar alveolectomy requires strict adherence to clearly defined postoperative instructions to minimize risks of complications such as alveolar osteitis, infection, prolonged bleeding or emphysema. Patients should bite on sterile gauze for 30 min after extraction to control bleeding, avoid rinsing the mouth during the first 24 h to preserve the blood clot, consume cool and soft foods and avoid spicy foods and hot beverages for 24–48 h. They should also refrain from smoking for 7 days after surgery to reduce the risk of emphysema [18].

This case underlines the importance of reinforcing the instruction to avoid nose blowing, as the patient’s unawareness of this risk contributed to the complication.

## 4. Conclusions

Although rare, cervicofacial emphysema can have serious and potentially life-threatening consequences, making prompt recognition of symptoms essential. Early detection is key in preventing further spread and development of complications such as mediastinitis, pneumothorax, cardiac tamponade, air embolism, and cardiac failure. We recommend including clear postoperative instructions following third molar alveolectomy that advise patients to avoid activities that increase tissue pressure, such as nose blowing, in the first few days after surgery. To our knowledge, this is the first reported case of nasopharyngeal emphysema associated with voice alteration after third molar alveolectomy. Although rare, this complication calls for awareness among dentists and oral surgeons; simple preventive advice can avert potentially serious outcomes. Early recognition by the oral surgeon allowed prompt management in our case, likely contributing to the favorable outcome.

## Figures and Tables

**Table 1:** Clinical course timeline.

Day 0	Alveolectomy of the Lower Right Third Molar
+1 h	Patient blows his nose and develops facial swelling and subcutaneous emphysema; prescription of oral antibiotics.
+6 h	Patient presents to the emergency department with an altered voice. CT scan is performed and cervicofacial and upper thoracic wall emphysema diagnosed. Patient is hospitalized and i.v. antibiotics administered.
day 1–5	Gradual improvement of symptoms.
day 5	Patient is discharged and oral antibiotics are prescribed for another seven days.
day 14	Follow-up examination reveals a full recovery.

## Data Availability

The original contributions presented in this study are included in the article. Further inquiries can be directed to the corresponding author.
